# Interplay of Na^+^ Balance and Immunobiology of Dendritic Cells

**DOI:** 10.3389/fimmu.2019.00599

**Published:** 2019-03-29

**Authors:** Patrick Neubert, Agnes Schröder, Dominik N. Müller, Jonathan Jantsch

**Affiliations:** ^1^Institute of Clinical Microbiology and Hygiene, University Hospital Regensburg, University of Regensburg, Regensburg, Germany; ^2^Department of Orthodontics, University Hospital Regensburg, University of Regensburg, Regensburg, Germany; ^3^Experimental and Clinical Research Center, A Joint Cooperation of Max-Delbrück Center for Molecular Medicine and Charité-Universitätsmedizin Berlin, Berlin, Germany; ^4^Max-Delbrück Center for Molecular Medicine in the Helmholtz Association, Berlin, Germany

**Keywords:** dendritic cells, Na^+^ balance, antigen-presentation, Nfat5, kidney, skin microenvironment

## Abstract

Local Na^+^ balance emerges as an important factor of tissue microenvironment. On the one hand, immune cells impact on local Na^+^ levels. On the other hand, Na^+^ availability is able to influence immune responses. In contrast to macrophages, our knowledge of dendritic cells (DCs) in this state of affair is rather limited. Current evidence suggests that the impact of increased Na^+^ on DCs is context dependent. Moreover, it is conceivable that DC immunobiology might also be influenced by Na^+^-rich-diet-induced changes of the gut microbiome.

Dendritic cells (DCs) represent important sentinel cells that continuously scan their microenvironment and play a key role in inducing immune responses and maintaining immunogenic tolerance [reviewed in (1–3)]. It is accepted that DCs are able to respond to a plethora of proteinaceous, lipid or carbohydrate molecules as well as nucleic acids via specialized receptors and signaling pathways [reviewed in (4–6)]. Recently, however, it emerged that the local Na^+^ electrolyte abundance impacts on innate and adaptive immune cell function and vice versa [reviewed in (7, 8)].

## Extrarenal Na^+^ Storage

In general, body Na^+^ and fluid homeostasis are known to be regulated in very narrow limits. Disturbing this balance by excessive dietary salt intake is linked to various diseases including hypertension and autoimmunity, which ultimately results in increased morbidity and mortality [reviewed in (9, 10)]. Traditionally, the kidney was seen as the sole organ that controls body salt content and fluid regulation. For that purpose, Na^+^ concentrations of about 400 mM can be reached at the renal loop bend accompanied by osmolalities of up to about 1,200 mOsm/kg in the renal medulla (11). The remaining extracellular body fluids are thought to readily equilibrate with plasma. Therefore, extra-renal regulation of total body and certain tissue Na^+^ content and concentration was largely ignored [reviewed in (7, 12–15)], even though evidence of interstitial salt storage was provided already in 1909, when chloride storage was found in the skin during pre-clinical studies (16, 17). Within the last twenty years, however, the interstitium of the skin has emerged as important organ involved in maintaining body Na^+^ balance. For instance chemical analysis in rodents revealed that the effective osmolyte concentration in skin tissue (i.e., skin (Na^+^+K^+^)/skin water) can reach levels of about 190 mM which is substantially higher than the effective osmolyte concentration in plasma of about 145 mM (18). Recent evidence using ^23^Na MRI and mathematical modeling demonstrate that very high Na^+^ concentrations are present at the epidermal and dermal junction zone (19, 20). Of note, chemical analysis of skin biopsies confirmed that the skin may serve as a Na^+^ buffer also in humans (21). This Na^+^-storage is reversible by dialysis (22, 23) and is able to strengthen the innate immune barrier by invigorating macrophage-dependent responses against intruding pathogens (24).

Elevated Na^+^ deposition is paralleled by changes in the gel-like cutaneous collagen matrix (25–27). Upon Na^+^-rich diets, there is an increased sulfation of glycosaminoglycan (GAGs) which might enable cutaneous Na^+^ storage [reviewed in (15)]. In addition to high Na^+^ containing diets (18, 27–29), it emerged that superficial skin infections (24) and chronic inflammatory processes (30) are able to trigger local Na^+^ accumulation. The mechanisms underlying both, the diet-dependent and diet-independent Na^+^ accumulation in the skin are, however, unknown. It is tempting to speculate that soluble or cell-bound mediators are able to modulate the GAG network's ability to serve as a negative charge capacitor facilitating local Na^+^ accumulation (27). Moreover, aldosterone and glucocorticoids may play an important role in this state of affair (31).

## Dendritic Cells as Potential Regulators of cutaneous Na^+^ stores

While the mechanisms that allow for local cutaneous Na^+^ accumulation remain elusive, depletion of mononuclear phagocytes using clodronate liposomes unraveled that these cells play an important role in regulating cutaneous Na^+^ stores (18, 28). In addition, targeting the osmoprotective transcription factor *nuclear factor of activated T cells 5* (*Nfat5*) in myeloid cells using Lyz2 (Lysozym2)/ LysM-Cre deleter mice revealed that this transcription factor plays an important role in sensing Na^+^-rich diet-induced local hypertonic environments (29). This myeloid cell specific osmoprotective response included the upregulation of the *Nfat5* target gene vascular endothelial cell growth factor C (*Vegfc*) which ultimately leads to lymphcapillary hyperplasia facilitating removal of Na^+^ from the skin (18, 29). Recent evidence also suggests that local Na^+^ storage additionally increases lymph flow in muscle and skin (32).

**Figure 1:**
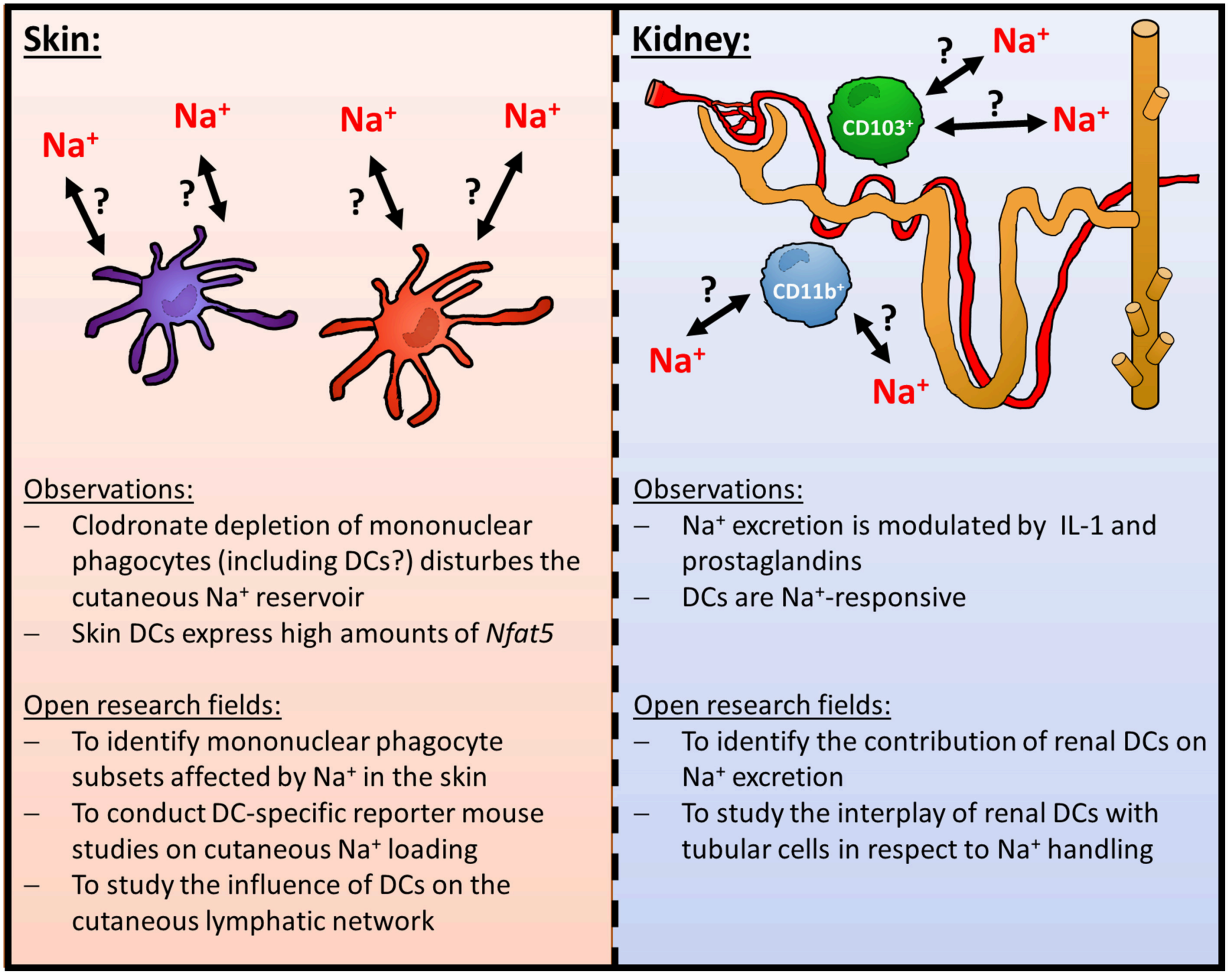
Role of DCs in homeostatic cutaneous and renal Na^+^-handling.

However, clodronate liposomes are known to deplete various mononuclear phagocytes in the skin including monocytes, macrophages and DCs (33). Moreover, although Lyz2 Cre primarily induces recombination in granulocytes, monocytes and macrophages, there is some recombination occurring in DCs (34, 35). In the Immgen Database (www.immgen.org), DCs, for instance, from skin draining lymph nodes (LN) (CD11c^+^, MHCII^hi^, Langerin^−^, CD11b^−^ CD103^−^ CD8a^−^ CD4^−^; CD11c^+^, MHCII^hi^, Langerin^−^, CD11b^+^ CD103^−^ CD8a^−^ CD4^−^; CD11c^+^, MHCII^hi^, Langerin^+^, CD11b^low^, CD103^+^, CD8a^−^, CD4^−^; CD11c^+^, MHCII^hi^, Langerin^+^, CD11b^+^ CD103^−^ CD8a^−^ CD4^−^) express very high *Nfat5* levels, suggesting that these cells might be involved in organization and regulation of cutaneous Na^+^ balance. To the best of our knowledge, the relative contribution of different mononuclear phagocyte subtypes including various DC subtypes in this state of affair is, however, unexplored. The use of novel DC- and macrophage-specific (transcriptional) reporter mouse strains and ablation strategies might be useful to uncover the relative contribution of distinct mononuclear phagocyte subtypes [reviewed in (36–38)]. It is likely that, in addition to macrophages, DCs might fulfill distinct tasks in regulating cutaneous Na^+^ balance. Recently, Randolph and colleagues demonstrated that lymphatic vessel permeability is controlled by DCs in a G protein-coupled homing receptor CCR7-dependent manner. Further analysis revealed that this task is fulfilled by IFN regulatory factor 4-positive DC subset (39). Taking these observations and the data from the Immgen database into account it is possible that DC-mediated regulation of the lymphatic vessels might be involved in facilitating the drainage of excess Na^+^ from cutaneous interstitial space (Figure 1).

## Dendritic cells as potential regulators of renal Na^+^ handling

In addition to regulating local Na^+^ balance in the skin, it is conceivable that DCs play a key role in orchestrating renal electrolyte handling. It is well-established that there is a dense network of mononuclear phagocytes including macrophages and DCs throughout the kidney. These cells play an important role in various inflammatory and fibrotic kidney injury models [reviewed in (40–43)]. Furthermore, they are able to change their shape and motility upon tissue damage (44, 45) and are involved in curtailing and/ or promoting inflammatory responses after various insults (46–52). Under *steady state*, the mononuclear phagocyte compartment of the mouse kidney mainly consists of CD103^+^ and CD11b^+^ renal mononuclear phagocyte subsets [reviewed in (41, 43, 53)]. The CD103^+^ mononuclear phagocytes are derived from *bona fide* DC precursors and these renal DCs play an important anti-inflammatory role upon renal damage (52, 54). The CD11b^+^ renal mononuclear phagocytes represent over 90% of the renal mononuclear phagocyte population and comprise DCs and macrophages [reviewed in (41, 43, 53)]. In contrast to the CD103^+^ renal mononuclear phagocytes/ DCs, the DC subset of these CD11b^+^ mononuclear phagocytes exerts proinflammatory functions (54). Of note, recent evidence using a transcriptional reporter mouse for DCs (zinc finger and BTB domain containing 46 [*Zbtb42*]-GFP; visualizing both CD103^+^ and CD11b^+^ DCs) demonstrates that CD103^+^ and CD11b^+^ renal DC subsets are round-shaped and located around blood vessels while in contrast counterintuitively most of the other renal mononuclear phagocytes (i.e., macrophages) are dendritically shaped (54).

While there is substantial evidence that these DCs are involved in inflammatory responses in the kidney it is currently unclear whether DCs contribute to the regulation of renal Na^+^ excretion. Recent data indicates that renal mononuclear phagocytes play an important role as accessory cells in regulating Na^+^ transport of renal tubular cells. Crowley and co-workers uncovered that IL-1-signaling modulates tubular Na^+^ excretion via mononuclear phagocytes in mice (55). Moreover, using a CD11b-Cre deleter mouse strain, Zhang et al. reported that prostaglandins derived from renal mononuclear phagocytes modulate the activity of renal Na^+^-Cl^−^ cotransporters (56). As the CD11b-Cre deleter mouse strain recombines in DCs as well (35), it is tempting to speculate that renal DCs are involved in this state of affair. This idea is further supported by the fact that murine DCs express specific molecules that facilitate the transport of Na^+^ and thus sensing of increased extracellular Na^+^ levels such as the sodium-potassium chloride cotransporter-1 (NKCC1), chloride cotransporter (NCC), the sodium-calcium exchanger (NCX) and the α and γ subunits of the epithelial sodium channel (ENaC) (57). Murine DCs are able to express gap junction proteins such as Connexin 43 (58), which are able to facilitate Na^+^ entry in addition to other molecules (59). It is tempting to speculate that DCs are able to form functional syncytial cell aggregates with tubular cells and thereby regulate renal Na^+^ handling. However, the contribution of these molecules in electrolyte physiology is unexplored and warrants further studies.

## Impact of Na^+^ on Dendritic Cell Immunobiology

**Figure 2:**
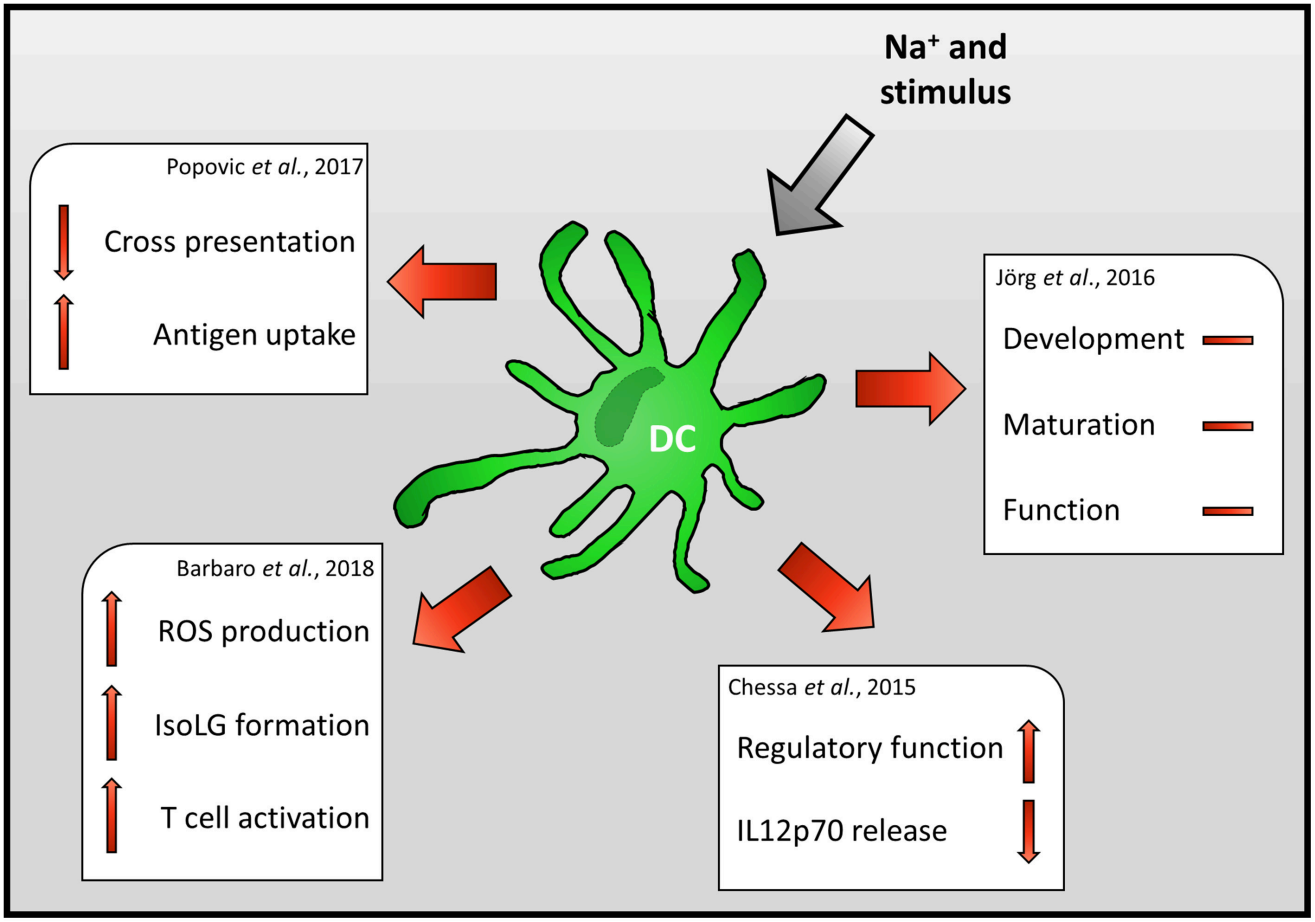
Impact of Na^+^ on DC immunobiology.

DCs might not only be important regulators of local Na^+^ balance. For instance there is robust evidence that increases in Na^+^ levels limits the anti-inflammatory capacity of macrophages while promoting their proinflammatory status (24, 60–65). Enhanced induction of proinflammatory macrophage activation required the activity of the osmoprotective transcription factor *Nfat5* (24, 64). Recently, Buxade et al. reported that *Nfat5* regulates the expression of MHCII molecules under standard cell culture conditions (i.e., normal salt conditions) and thereby regulates CD4^+^ T cell responses (66). This regulatory circuit only operates in macrophages but not in DCs (66). Surprisingly, the impact of increased Na^+^ levels on DC immunobiology has been studied in less detail and the data available are controversial (Figure 2).

Jörg et al., for instance, reported that high Na^+^ levels do not impact the generation, maturation or function of mouse DCs but rather directly impact on T cells (67). In contrast to these findings, Chessa et al. demonstrate that increasing extracellular Na^+^ levels, found in the renal medulla during DC development, skews murine DCs to a macrophage-like regulatory phenotype and suppresses the release of the Th1 priming cytokine IL-12p70 (68).

In line with this, Popovic et al. reported that the ability of mouse DCs to cross-present the model antigen ovalbumin is severely impaired (69). Decreased cross-presentation was recorded despite enhanced antigen uptake, processing, and presentation. Of note, increased Na^+^ levels resulted in enhanced expression of co-inhibitor and co-stimulatory molecules. Using knock out strategies and blocking antibodies the authors exclude that enhanced expression of co-inhibitory/ -stimulatory molecules or reduced production of IL-12 underlies this phenotype. The authors provide evidence that the suppressive effect of high salt conditions (HS) on cross-presentation is dependent on TIR-domain-containing adapter-inducing interferon-β (TRIF) regulated process. However, the TRIF-dependent mechanism that ultimately results in impaired cross-presentation requires further investigation (69). Recently, Zhang et al. reported that exposure of virally infected mouse macrophages to increased Na^+^ levels boosts the release of Type 1 interferon (65). Since TRIF and type 1 interferon production are intertwined [reviewed in (70)] and type 1 interferon signaling has the potential to inhibit antigen-presentation (71), it is conceivable that exposure to increased Na^+^ levels triggers an overshooting type 1 interferon response which ultimately inhibits cross-presentation by DCs.

In line with enhanced degradative activity of DCs upon HS exposure (69), Barbaro et al. found that increasing extracellular Na^+^ levels result in enhanced ROS production and formation of isolevuglandin (IsoLG)-protein adducts in mouse DCs. However, in contrast to the study using the model antigen ovalbumin, Barbaro et al. reported increased frequencies of IFN-γ and IL-17 producing T cells after co-incubation of DCs with T cells. Moreover, transfer of DCs exposed to high Na^+^ environments, increased the blood pressure of mice subjected to low levels of angiotensin II (57). These findings suggest that increased local Na^+^ levels enhance the inflammatory potential of DCs and, thus might propagate inflammatory circuits that ultimately result in arterial hypertension and cardiovascular death.

Of note, increases in dietary Na^+^ might not only directly influence the immunobiology of dendritic cells. Recently, Wilck et al. demonstrated that dietary high salt conditions change the composition of the microbiome by removal of *Lactobacillus murinus* (72). Depletion of *Lactobacillus* was accompanied by reduction of the tryptophan metabolites such as indole 3-lactic acid (ILA) and indole 3-acetic acid. Increased levels of ILA directly inhibit the proliferation of T_H_17 cells *in vitro* (72). In addition, it is possible that these tryptophan degradation products are impacting on gut dendritic cells, which in turn orchestrate e.g., regulatory T cell, T_H_22 and T_H_17 effector cell balance (73, 74). In line with this, there are several reports that Na^+^-rich diets increases the production of cytokines that are key players in screwing the induction of T_H_1 and T_H_17 cells in inflamed gut tissue such as *Il12b* and IL-23 (75, 76).

## Conclusion

Na^+^ availability emerges as a new factor of tissue microenvironment which on the one hand is regulated by immune cells and on the other hand is able to impact on their immunological function. In contrast to macrophages, our knowledge regarding DCs is rather limited. Current evidence suggests that the impact of increased Na^+^ levels on DCs is context dependent. However, the role of DCs in regulating local Na^+^ stores is unexplored and warrants further studies.

## Data Availability

Publicly available datasets were analyzed in this study. This data can be found here: “www.immgen.org.”

## Author Contributions

PN and JJ: conception and writing of the manuscript. AS and DM: contributed to the writing.

### Conflict of Interest Statement

The authors declare that the research was conducted in the absence of any commercial or financial relationships that could be construed as a potential conflict of interest.
